# Concurrent Wounded Glioma Syndrome and Distant Wounded Glioma Syndrome Following a Gross Total Resection of Glioblastoma: A Case Report

**DOI:** 10.7759/cureus.48915

**Published:** 2023-11-16

**Authors:** John Michael V Calubayan, Paul Vincent A Opinaldo

**Affiliations:** 1 Center for Neurological Sciences, Quirino Memorial Medical Center, Quezon City, PHL

**Keywords:** high-grade glioma (hgg), glioma surgery, glioblastoma, distant wounded glioma syndrome, wounded glioma syndrome

## Abstract

Surgery is the initial form of treatment for glioblastoma, and a maximum resection without impairing neurological function improves survival. Wounded glioma syndrome (WGS) is a clinical picture observed after the resection of high-grade tumors. This syndrome, developing within hours to a few days after glioma surgery, is characterized by hemorrhage into the postoperative cavity and cerebral edema and at times occurs in areas distant from the site of the resection, i.e., distant wounded glioma syndrome (DWGS). We report a case of a 70-year-old male presenting with acute-onset left leg weakness, with a large peripherally enhancing mass with central non-enhancement suggestive of necrosis in the right frontal lobe. A gross total resection of the tumor was done, and the histopathologic evaluation verified the diagnosis of glioblastoma World Health Organization (WHO) grade IV. During the postoperative period, he was drowsy and was able to move his right extremities. He had a series of generalized tonic-clonic seizures three hours after the operation. After eight hours, the patient became comatose with signs of increasing intracranial pressure. A cranial computed tomography (CT) scan revealed diffuse cerebral edema and hemorrhage into the operative site in the right frontal lobe, as well as subarachnoid hemorrhages in the bilateral frontoparietal sulci. There were also small hemorrhages seen in the left caudate head, midbrain, and left hemipons. Death occurred the following day. This case report demonstrates an unusual case of a WGS with a concurrent DWGS in the brainstem after a gross total resection of a frontal lobe glioma. This case shows a significantly uncommon sequela that a patient undergoing glioma surgery can present, leading to rapid deterioration and death. Resection of a glioma carries a significant risk, and its impact in the immediate postoperative period merits evaluation when planning perioperative management, taking prompt action if these syndromes occur.

## Introduction

Glioblastomas (World Health Organization (WHO) grade IV) are the most prevalent type of malignant primary brain tumor, accounting for the highest mortality rate among patients with primary brain tumors. They are particularly aggressive and treatment-refractory, with unfavorable prognosis (the five-year survival rate is between 1% and 19%, depending on age, pathology, and extent of resection) [1,2]. Although accounting for only 1.4% of all cancer cases, this type of cancer is responsible for 2.9% of all cancer-related deaths [1,2]. Patients and survivors of brain tumors have a higher risk of experiencing suicidal ideation and attempts than the general population [3]. Particularly, among different types of brain tumors, a glioblastoma diagnosis was shown to be associated with an even greater risk of suicide [3]. When a patient presents with a clinical and radiological picture suggestive of a high-grade glioma (defined as WHO grade III or IV), the current guidelines recommend, whenever possible, a maximal safe resection as first-line treatment [1,2,4]. Wounded glioma syndrome (WGS) is a known postoperative complication observed after a biopsy or a total or partial resection of a high-grade glioma [5]. This syndrome, developing within hours to days after glioma surgery, is characterized by hemorrhage into the surgical bed and cerebral edema and at times occurs in areas remote from the site of the resection or biopsy, hence, the term distant wounded glioma syndrome (DWGS) [5-9]. There is still very little literature on WGS and DWGS, and these have been limited only to case reports [5-9]. We report a rare case of a patient with a simultaneously occurring WGS and DWGS after undergoing a gross total resection of a glioblastoma multiforme (GBM).

This case report was previously presented as a poster at the 2023 Society for Neuro-Oncology (SNO)/American Society of Clinical Oncology (ASCO) Central Nervous System (CNS) Cancer Conference on August 10-12, 2023.

## Case presentation

Clinical course and radiological examinations

The patient was a 70-year-old Filipino male with an initial Karnofsky Performance Status (KPS) score of 100. He was independent in all activities, with no prior noticeable weakness, no behavioral or mood changes, memory lapses, or disorientation, and no sleep disturbances. He had no other known comorbidities aside from controlled hypertension, for which he was not on any medication. He primarily presented with acute-onset weakness of the left lower extremity, which was noted to progress over one day with no episodes of resolution. Over three weeks, the left lower extremity weakness was persistent with no associated numbness, headache, nausea or vomiting, blurring of vision or diplopia, dysarthria, facial asymmetry, changes in sensorium or behavior, seizures, or involuntary movements. His KPS score decreased to 70 before the consultation. The neurological examination showed the weakness of only the left lower extremity and revealed the presence of several frontal release signs, including glabellar tap, snout, palmomental, grasp, and left Babinski reflexes. He had normal sensory, cranial nerve, brainstem, and cerebellar functions.

**Figure 1:**
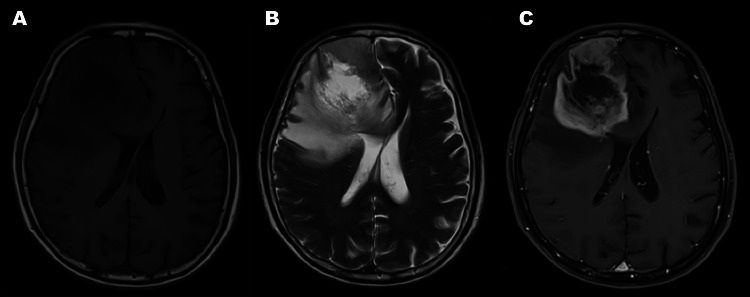
Preoperative MRI (A) Axial T1-weighted MRI showed an irregularly shaped hypointense lesion within the white matter of the right frontal lobe, surrounding a more hypointense core, which was hyperintense on T2-weighted imaging (B), with surrounding vasogenic edema, which exerts a mass effect to the adjacent medial frontal gyri and the frontal horn of both lateral ventricles. (C) A contrast study showed a sizeable mass exhibiting peripheral enhancement and central non-enhancement, which was indicative of necrosis. MRI: magnetic resonance imaging

Magnetic resonance imaging (MRI) (Figure 1) showed a large peripherally enhancing mass with central non-enhancement, indicative of necrosis, in the right frontal lobe. It was surrounded by moderate edema and exerted a mass effect on the adjacent medial frontal gyri and the frontal horn of both lateral ventricles. He was started on dexamethasone for medical decompression and was scheduled to undergo craniotomy for tumor resection after three weeks. The institution's inability to offer awake craniotomy precluded the execution of the aforementioned procedure.

**Figure 2:**
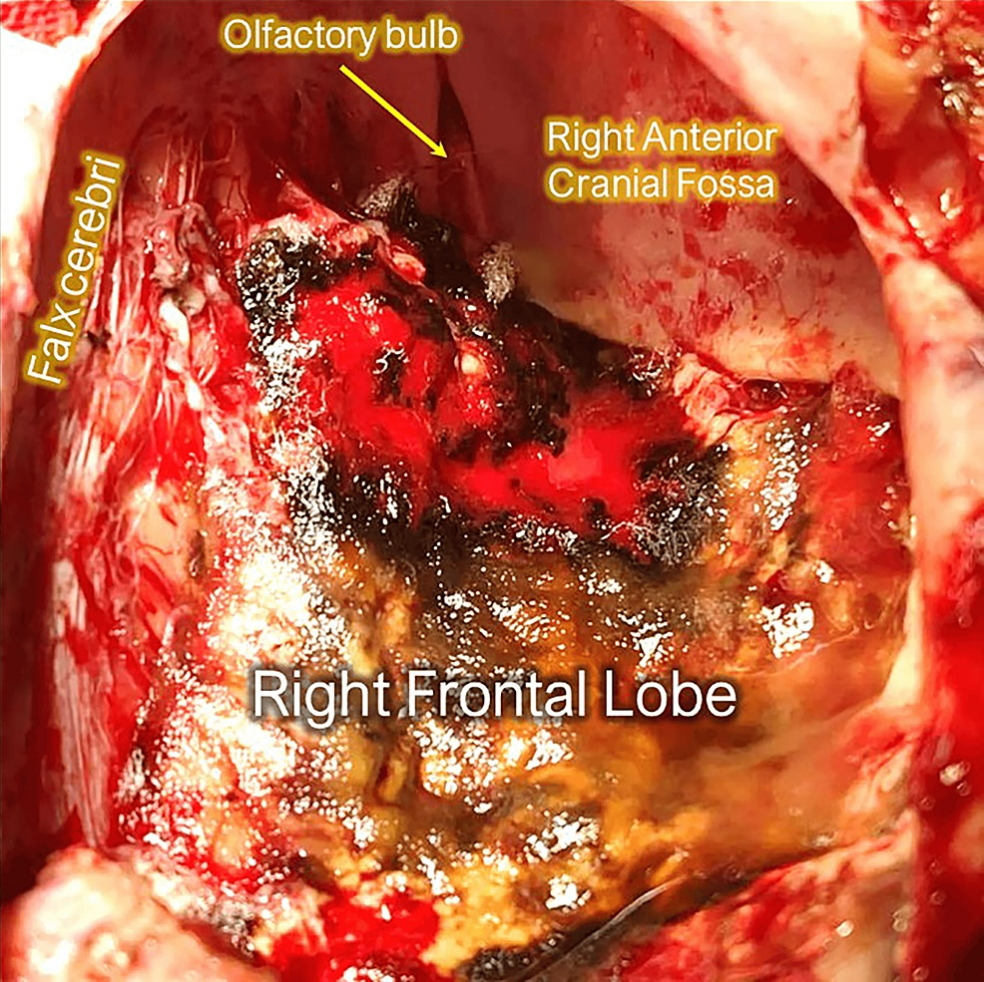
Intraoperative image after gross total resection of the tumor Superior view of the right anterior cranial fossa showing the cavity anterior to the right frontal lobe where the tumor was removed.

The patient underwent a right frontal craniotomy for gross total resection of the tumor (Figure 2). The administration of 5-aminolevulinic acid (5-ALA) facilitated a more precise determination of the extent of tumor resection. The surgical procedure lasted for seven hours, with an estimated blood loss of 1 L. To address this, two units of packed red blood cells were transfused. Notably, no complications occurred during the operation.

**Figure 3:**
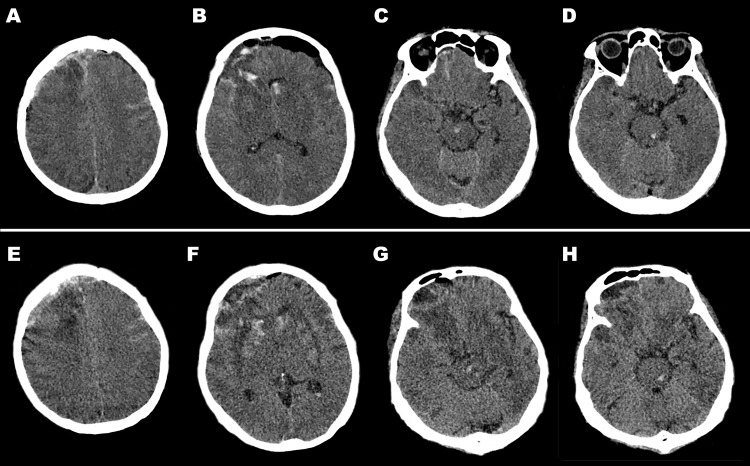
Postoperative CT scan (A-D) Axial CT scan showed subarachnoid hemorrhages in the bilateral frontoparietal sulci and subdural hemorrhage in the right frontal convexity (A), hemorrhage in the surgical bed in the right frontal lobe, and acute hemorrhages in the left caudate head (B), midbrain (C), and left hemipons (D). There was also diffuse cerebral edema, with effacement of the bilateral frontal horns of the lateral ventricles and right cerebral cortical sulci. (E-H) A follow-up CT scan on the first postoperative day showed progression of cerebral edema, with the brain parenchyma now occupying the previous postoperative cavity in the right anterior cranial fossa and the frontal horns of both lateral ventricles (more in the right) still compressed. An increase of the subdural hemorrhage in the right frontal convexity is noted, with no change in the subarachnoid hemorrhages in the bilateral frontoparietal sulci (E). There were hemorrhages in the bilateral caudate and lentiform nuclei (F), with no significant change in the amount of hemorrhages in the midbrain (G) and left hemipons (H). CT: computed tomography

Following the surgical procedure, he experienced a state of lethargy and exhibited right-sided hemiparesis. He had a series of generalized tonic-clonic seizures three hours after the operation, initially resolved with the administration of intravenous diazepam. He was then started on a continuous midazolam drip for seizure control. A computed tomography (CT) scan (Figure 3A-3D) revealed diffuse cerebral edema, subdural hemorrhage in the right frontal convexity, and subarachnoid hemorrhages in the bilateral frontoparietal sulci. There were also hemorrhages in the surgical bed in the right frontal lobe and in the left head of the caudate nucleus, midbrain, and left hemipons. Medical decompression using mannitol was done. After eight hours, the patient became comatose with signs of increasing intracranial pressure. Maximal conservative therapy was unable to ameliorate the clinical state. A follow-up CT scan done the following day (Figure 3E-3H) showed an increase in cerebral edema and subdural hemorrhage in the right frontal convexity. There were hypodense changes involving the bilateral lentiform and caudate nuclei, with interspersed inhomogeneous hyperdensities, denoting subacute infarction with hemorrhagic transformation. There was no change in the subarachnoid hemorrhages in the bilateral frontoparietal sulci and the hemorrhages in the left caudate, midbrain, and left hemipons. The results of the CT angiogram and venogram did not reveal any significant findings, indicating a normal state of the examined vessels. Despite maximal conservative management, the patient expired on the same day.

Histopathology

**Figure 4:**
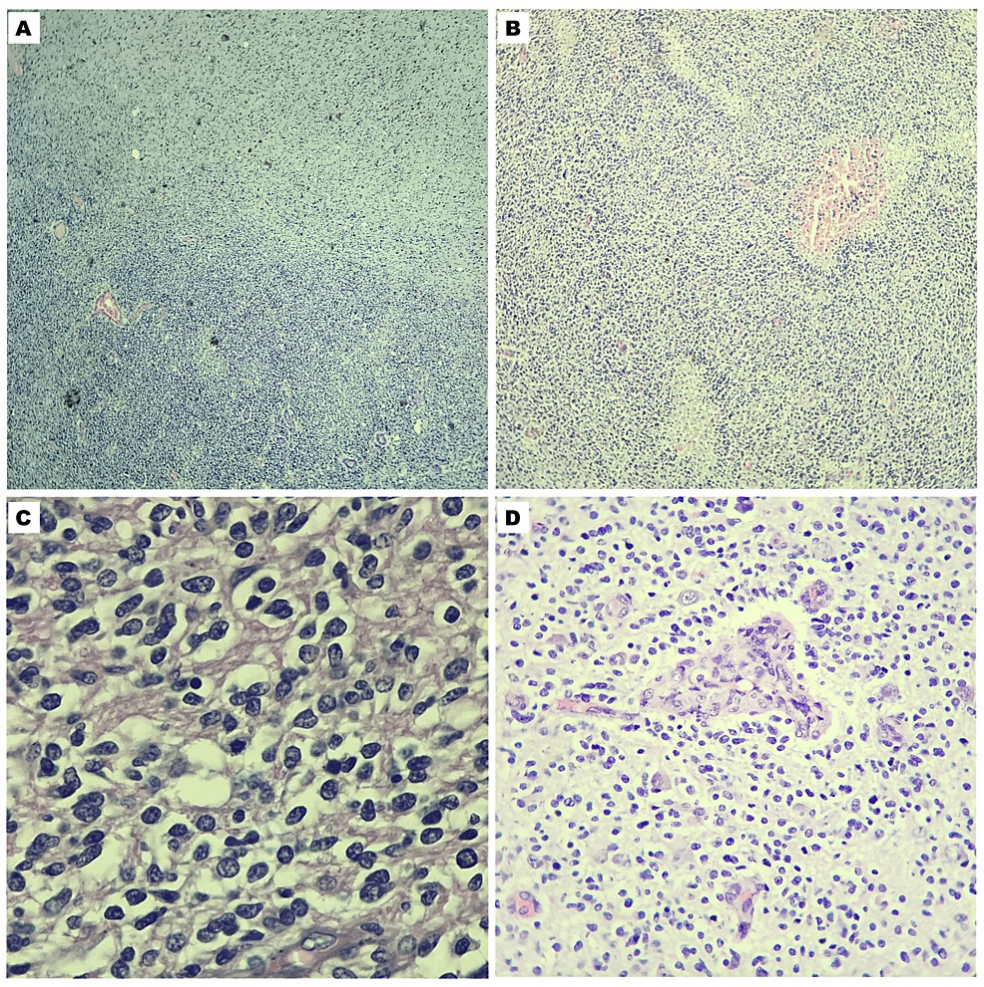
Microsections of the right frontal lobe tumor The hypercellular neoplasm that infiltrates the cerebral parenchyma (A), with areas of pseudopalisading necrosis (B). The neoplasm consists of round to spindle cells with prominent nucleoli, scanty cytoplasm, and brisk mitotic activity (C). Vascular changes took the form of endothelial cell proliferation (D).

Intraoperatively, the right frontal lobe tumor appeared as a soft gray-tan intra-axial mass with irregular borders. Microsections (Figure 4) show a hypercellular neoplasm that infiltrates the cerebral parenchyma. The neoplasm comprises round to oval cells with scanty cytoplasm, with brisk mitotic activity, necrosis, and endothelial cell proliferation. The histopathologic evaluation was compatible with high-grade glioma, favoring a diagnosis of glioblastoma, not otherwise specified (NOS), WHO grade IV. Immunohistochemistry was not performed due to the demise of the patient, and consent for postmortem examination was withheld by the family.

## Discussion

We report a case of an elderly male who underwent a gross total resection of glioblastoma of the right frontal lobe. During the postoperative period, he experienced seizures and signs of increased intracranial pressure, leading to a coma. Following the cranial surgery, the subsequent CT scans revealed the presence of extensive cerebral edema and hemorrhages. These hemorrhages were found not only at the surgical site located in the right frontal lobe but also in several remote regions. The postoperative complications described are consistent with the findings of WGS and DWGS, as discussed in the limited available literature on these clinical entities [5-9]. The patient in this report is unique because the WGS and DWGS developed simultaneously, whereas the published case reports showed that these complications occur independently.

WGS is recognized as a postoperative complication that may arise after a tumor has been completely or only partially removed. It involves bleeding into the tumor bed, which may occur soon after surgery despite best efforts to obtain hemostasis throughout the operation [5-7]. The current case reports indicate that WGS can occur following tumor resection or biopsy. One report highlights two cases of malignant cerebral edema, a rare and life-threatening manifestation of WGS, in adult male patients who underwent open and stereotactic biopsy for gliomas. The first case presented with severe morbidity, while the second case resulted in in-hospital mortality [6]. One pediatric patient showed symptoms of intracranial hypertension after partial surgical resection of anaplastic astrocytoma, with a follow-up CT scan revealing bleeding in the surgical bed and significant perilesional edema [7]. In addition, DWGS has been used to describe hemorrhage in areas not directly manipulated during surgical procedures [5,6]. One case report documented two middle-aged males with GBM without known risk factors who presented with postoperative hemorrhaging [5]. These patients underwent image-guided gross total resection of the lesion, only to develop hemorrhage at distant tumor sites that were not directly manipulated during the surgical procedures. The remote hemorrhage was not directly related to the surgical procedures, yet it caused new neurological deficits that led to severe morbidity [5]. An additional report examining DWGS consequent to stereotactic biopsy of glioblastoma revealed the presence of small, distant hemorrhaging within a tumor nodule far removed from the initial biopsy site [8]. In the cases at hand, performing total or partial resection of gliomas, as well as stereotactic or open biopsy, has resulted in bleeding at the site of the operation as well as in distant areas. In some instances, this has led to the development of malignant brain edema after surgery.

Pathophysiology

Glioblastoma is characterized by its ability to create extensive networks of microvasculature through overactivated angiogenesis [10]. In addition, the overexpression of chemokine receptors on glioma cells has been linked to perivascular invasion [11]. This results in significantly elevated levels of vascular endothelial growth factor (VEGF) and chemokines that lead to the disruption of the blood-brain barrier (BBB) [10]. The rapid cellular proliferation and angiogenesis lead to the development of highly vascular malignant tissue, which is susceptible to spontaneous bleeding. The surgical disruption of this richly vascularized tissue is a contributing factor to the development of WGS [5]. Disruption of these barrier properties also leads to vessel permeability and leakage of plasma into the tumor tissue, which gives rise to cerebral edema and mass effect [10]. Local hemodynamic changes occur due to peritumoral edema. This edema causes a decrease in regional blood flow resulting in vasoparesis and loss of autoregulation [5]. After the removal of a large tumor, there may be a sudden increase in blood flow into the already edematous area of the brain secondary to the normal perfusion pressure breakthrough (NPPB) [5]. This phenomenon is similar to that observed after the resection of arteriovenous malformations [5]. The resection of the sizable tumor mass in this particular case could have resulted in local bleeding within the tumor bed. However, this observation does not account for the reason causing bleeding in locations remote from the site of the craniotomy.

Complete resection of GBM poses a significant challenge due to the tendency of glioma cells to migrate from the primary tumor and potentially infiltrate the contralateral hemisphere [10]. There are several well-described causes of remote site hemorrhage (RSH) that could account for the observed DWGS [12]. A comprehensive analysis conducted over 11 years on 4,992 intracranial procedures revealed that postoperative bleeding occurred in 0.8% of the patients [13]. Coagulopathy, predominantly drug-induced, and hypertension were determined as the potential precipitating factors related to postoperative hemorrhaging [14]. Excessive drainage of cerebrospinal fluid (CSF) has been postulated as a potential cause of hemorrhage following craniotomy, particularly in the context of cerebellar hemorrhage after supratentorial surgery [5,12]. It seems that dural opening, intraoperative and/or postoperative CSF loss, patient position during operation, preoperative antithrombotic usage, coagulation abnormalities, intraoperative elevation of systolic blood pressure, and impaired venous drainage are proposed to have a causative role in RSH [12]. Excessive cerebrospinal fluid (CSF) drainage or retraction-induced mechanical shifting of the brain may impede venous drainage and lead to hemorrhage [5]. The patient under consideration was deemed physically fit, with controlled hypertension, and received a cardiopulmonary clearance for the intended surgical procedure. Our findings indicate that he did not experience hypertension during the perioperative period, and a thorough review of the anesthesia record revealed no evidence of transient blood pressure changes throughout the surgical procedure. No antithrombotic agents were given to the patient, and his prothrombin time, partial thromboplastin time, platelet count, and bleeding time were normal. There was no excessive drainage of cerebrospinal fluid, and he did not undergo any lumbar or ventricular drainage during the tumor removal surgery. Based on the available evidence, it is suggested that hypertension, coagulopathy, and CSF drainage are improbable factors contributing to the postoperative hemorrhaging in this case.

The DWGS seen in the patient is most likely attributable to tumor-induced coagulopathy. This suggests that changes in coagulation caused by either the tumor or the surgery contribute to the formation of multiple postoperative intracranial hemorrhages. These hemorrhages are observed bilaterally, affecting not only the site of the tumor resection but also other areas of normal brain tissue [5]. During surgery, the patient can develop a local hyperfibrinolysis from a pronounced local release of fibrinolytic substances into the systemic circulation [14]. The surgical resection of a large, deficit-producing tumor can create an immediate systemic coagulation dysfunction. This results in a fibrinolytic mechanism akin to disseminated intravascular coagulation (DIC), leading to decreased platelet counts, elevated levels of D-dimer, and diminished maximum clot strength. This cascade of events ultimately leads to a hypocoagulable state at distant tumor sites [5,14]. Resection of a primary lesion leads to a significant increase in blood flow, particularly in remote tumor areas. The presence of peritumoral edema in these regions renders the tissue susceptible to vasoparesis. This systemic hypocoagulable state, combined with the increased blood flow into the leaky, hemorrhage-prone vessels of the remote tumor nodules, increases the likelihood of postoperative hemorrhaging [5].

The primary objective of glioblastoma surgery is to achieve maximal safe resection of the enhancing solid tumor while preserving neurological function [1,2,4]. The mortality rate after craniotomy for glioma has decreased over the past few decades owing to the advancement in imaging modalities and intraoperative adjuncts [10]. Despite being a potentially lifesaving procedure, intracranial surgery to remove tumors is associated with a high risk of complications. The overall complication rates range from 9% to 40%, with 3.2% of all glioma patients experiencing intracranial hemorrhage within seven days of surgery [9]. The integration of several techniques, including intraoperative MRI, neuronavigation, ultrasonography, and fluorescence-guided surgery, has led to safer and more comprehensive surgical resection, resulting in improved survival and postoperative outcomes [10].

From a clinical perspective, it would be prudent to consider both WGS and DWGS as potential surgical complications when planning the best management approach for gliomas. It is important to discuss the possibility of these complications with patients before deciding on surgery. To mitigate neurological complications, surgical intervention should aim for complete removal of lesions situated in areas where excision would not cause further neurological impairment. Nevertheless, complete tumor resection may not be feasible with distant lesions in eloquent brain regions. In such instances where reducing novel neurological deficits is critical while simultaneously achieving the maximum extent of resection, the preferred approach is awake craniotomy with intraoperative electrical stimulation mapping [10]. It is crucial to consider these limitations when planning treatment options and to explore alternative strategies for managing such cases. The available literature shows no defined protocol for managing WGS or DWGS. In the previous case reports, the patients were managed on a case-to-case basis, according to the patient's neurological status and the volume and site of the bleeding and edema. The clinical challenge remains how to treat the local and distant hemorrhaging and the concomitant malignant edema. Biopsy of brain tumors has previously been considered sufficient in cases where at least subtotal resection cannot be achieved, as partial resection has not been shown to impact survival significantly. However, even partial tumor resection and stereotactic or open biopsy of gliomas have been shown to lead to hemorrhage and malignant postoperative cerebral edema [6-8]. While it may not be cost-effective to routinely monitor for this rare complication, closely monitoring coagulation studies and thromboelastography (TEG) during surgery can help neurosurgeons identify early signs of DIC-like changes. This allows for prompt treatment during the procedure, which can prevent the development of WGS and/or DWGS. Studies are still required to estimate the true impact of the WGS and DWGS since much of their pathophysiology remains uncertain.

## Conclusions

In the present case, the gross total resection of a large GBM tumor has led to a fatal combination of WGS and DWGS. Hemorrhage occurring into and remote from the surgical resection site is a rare yet potentially serious complication that warrants attention when postoperative deterioration occurs. This is especially critical when the presenting symptoms are not consistent with the area that underwent surgery. Despite extensive research into the pathobiology of GBM and the remarkable progress that has been made in its management, treating this typically fatal cancer remains a challenge. A thorough assessment of coagulation and TEG studies done perioperatively can be instrumental in preventing these potential complications. Surgeons and patients must consider WGS and DWGS as possible complications before deciding to perform a particular surgical procedure. We suggest that a discussion of these possible complications, occurring independently or concurrently, be included as part of the informed consent for the surgical procedure. These syndromes and their implications and repercussions in the immediate postoperative period must be reviewed to evaluate the risks and benefits of surgery and avoid fatal outcomes.
